# Supplementary material for: TRIM28 protects TRIM24 from SPOP-mediated degradation and promotes prostate cancer progression
Source: Nat Commun. 2018 Nov 27;9:5007. doi: 10.1038/s41467-018-07475-5 (PMC6258673; doi:10.1038/s41467-018-07475-5)
Supplement: Supplementary file 1 — Supplementary Information [file 41467_2018_7475_MOESM1_ESM.pdf]

SUPPLEMENTARY INFORMATION

TRIM28 protects TRIM24 from SPOP-mediated degradation and promotes prostate cancer progression

Ka-wing Fong et al.

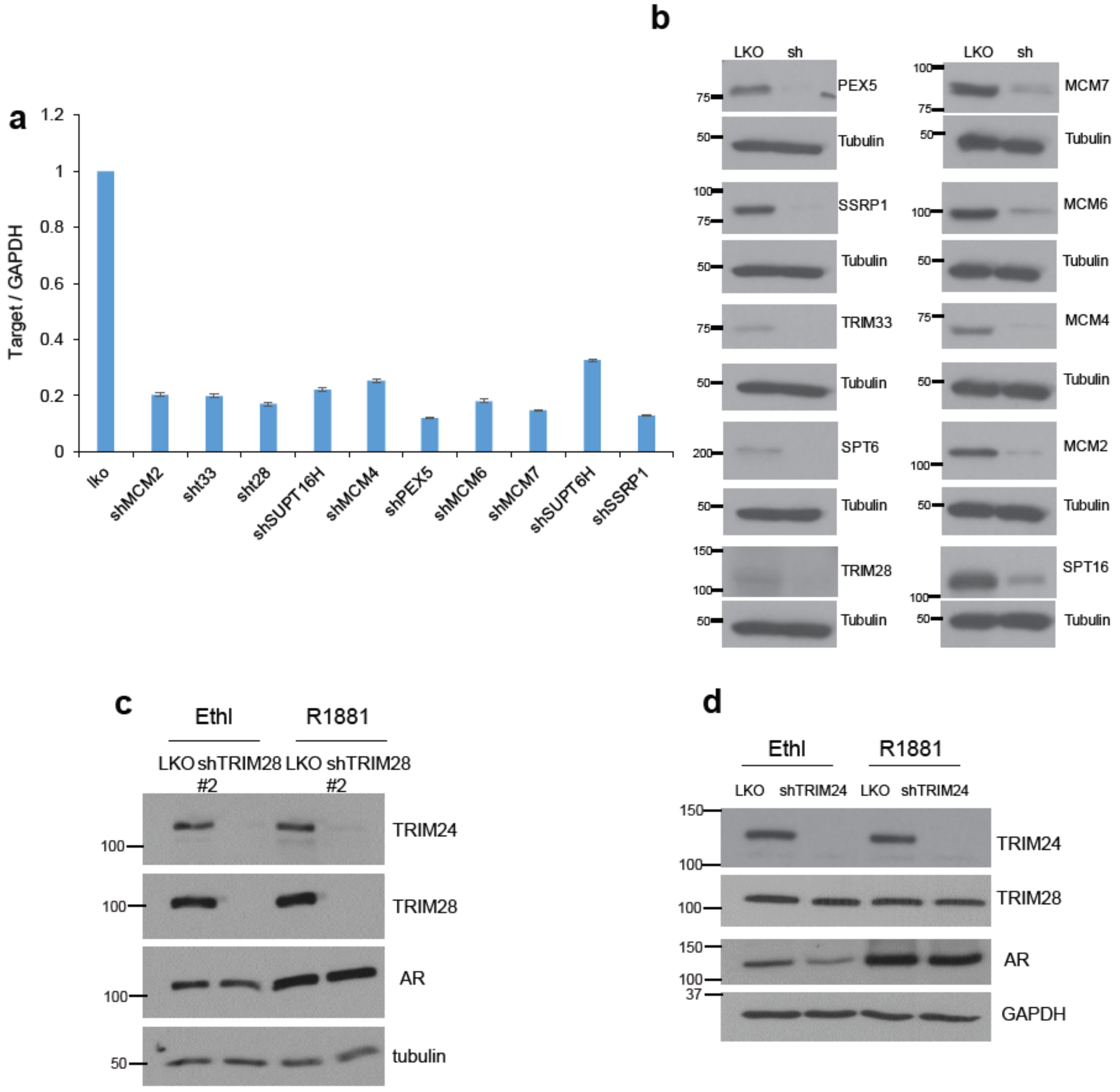

Supplementary Figure 1 (related to Figure 1). Systematic screening of TRIM24-interactors identifies TRIM28 as a positive regulator of TRIM24 protein stability.

(a) RNA were extracted from LNCaP cells infected with indicated shRNAs. QRT-PCR analysis was performed using corresponding primer sets and normalized to GAPDH. Data shown is mean (±SEM, *n*=3).

(b) Protein lysates were harvested from LNCaP cells infected with indicated shRNAs. Immunobloting was performed using antibodies corresponding to each protein.

(c) TRIM28 stabilizes TRIM24 protein in LNCaP cells. LNCaP cells expressing shCtrl or a second shRNA against TRIM28 were hormone starved for 3 days and treated with ethanol or 1nM R1881for 48h. Protein lysates were harvested and subjected to immunoblotting analysis.

(d) TRIM24 is not required for TRIM28 protein stability. LNCaP cells infected with lentiviral supernatant containing LKO and shTRIM24, respectively for 4 days. Protein lysates were then harvested and subjected to immunoblotting.

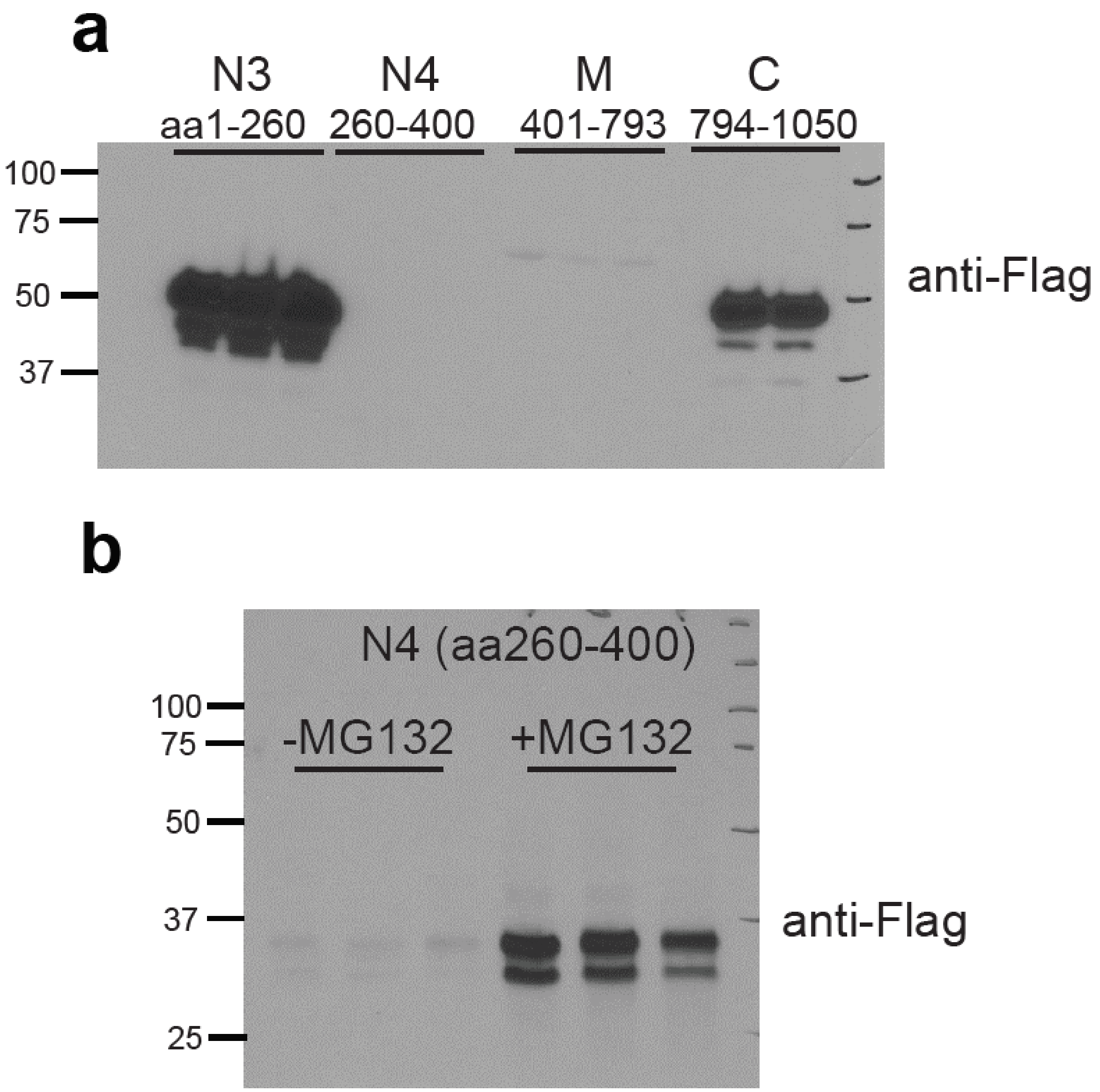

Supplementary Figure 2 (related to Figure 2). Some N-terminal TRIM24 deletion constructs were degraded by proteasome-mediated degradation.

(a) Various TRIM24 fragments with an SFB tag were transiently expressed in HEK293T cells. Protein lysates were collected and western blot was performed to detect Flag-tag protein.

(b) TRIM24 fragments were regulated by proteasome degradation. TRIM24 N4 (aa260-400) was expressed in HEK293T and treated with DMSO or 10μM MG132 for 8hrs. Immunoblot was performed to detect Flag-tag proteins in protein lysates.

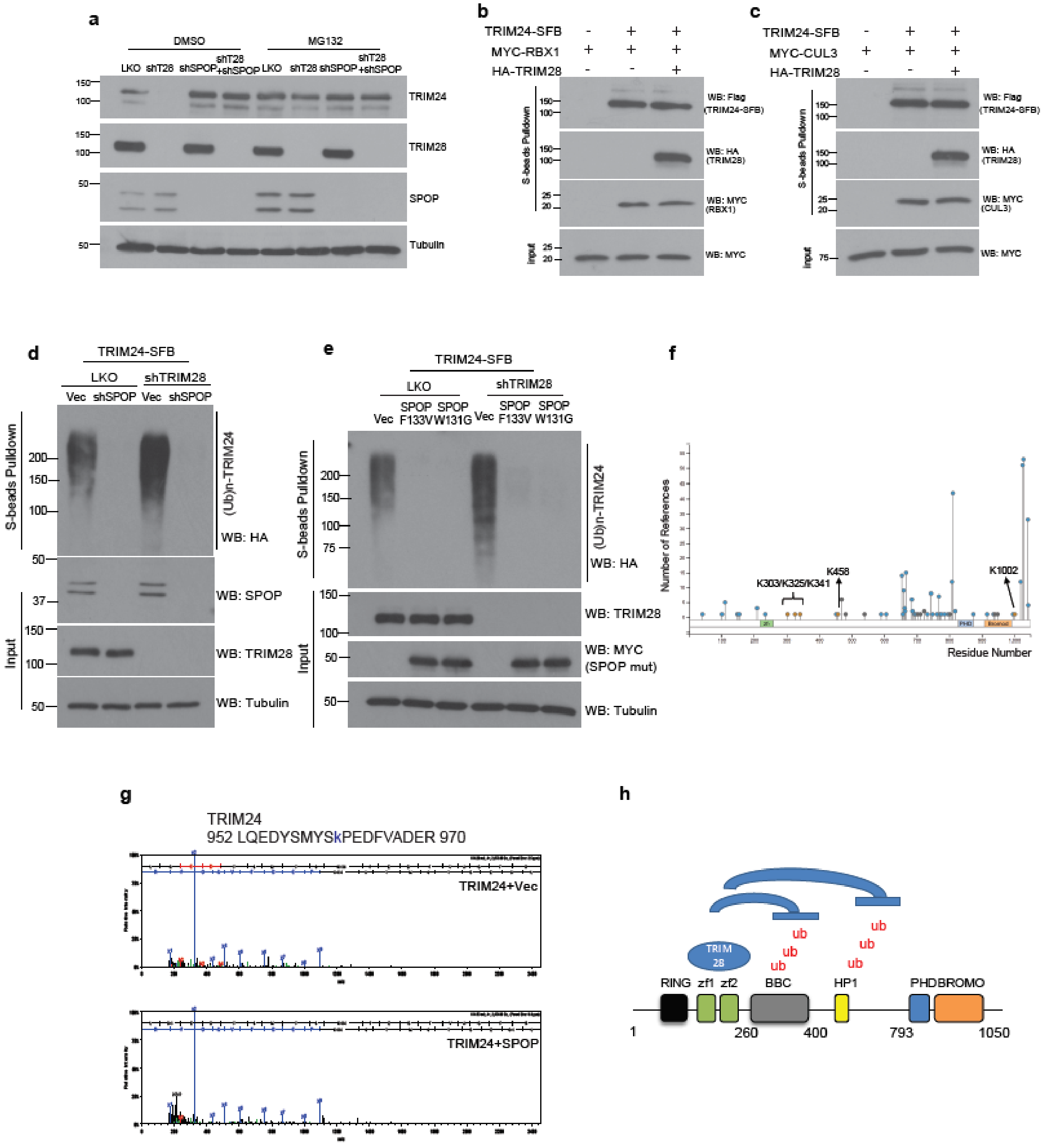

Supplementary 3 (related to Figure 3). TRIM28 inhibits SPOP-mediated ubiquitination and degradation of TRIM24.

(a) TRIM28 protects TRIM24 from SPOP-driven proteasome-mediated degradation. LNCaP cells expressing shCtrl, shTRIM28, shSPOP or shTRIM28+shSPOP were treated with DMSO or 10uM MG132 for 8 hrs and protein lysates were subjected to western blot analysis using indicated antibodies.

(b-c) TRIM28 does not affect RBX1 or CUL3 binding to TRIM24. HEK293T cells co-transfected with TRIM24-SFB and empty vector, Myc-RBX1 **(b)** or Myc-CUL3 **(c)** and HA-TRIM28 were treated with 20uM MG132 for 24 hrs and then harvested. S-beads were used to pull down TRIM24-SFB from cell extract, bound proteins were analyzed by immunoblotting using indicated antibodies.
(d) TRIM28 knockdown promotes SPOP-mediated ubiquitination of TRIM24. S-beads pull down was performed using protein extracts of MG132-treated LNCaP cells expressing TRIM24-SFB and HA-Ub were infected with pLKO, shSPOP, shTRIM28 or shTRIM28+shSPOP. The enriched protein complexes were analyzed by immunoblotting.
(e) SPOP mutants abolishes TRIM24 ubiquitination. LNCaP cells were transfected with TRIM24-SFB, HA-Ub, and Myc-SPOP F133V or SPOP-W131G in control or TRIM28-depleted condition. After 4hrs of 20uM MG132 treatment, cells were harvested and cell lysates were subjected to S-beads pull down followed by immunoblotting.
(f) Identification of potential TRIM24 ubiquitination sites. TRIM24 protein post-translational modification was profiled by PhosphoSitePlus (www.phosphosite.org).
(g) Protein ubiquitination analysis by mass spectrometry. TRIM24-SFB were co-transfected with vector or SPOP. Flag-IP proteins were analyzed by mass spectrometry and scanned for ubiquitination sites. Ubiquitinated K961 is highlighted in blue color.
(h) TRIM28 binds to TRIM24 protein to protect it from ubiquitination by wildtype SPOP.

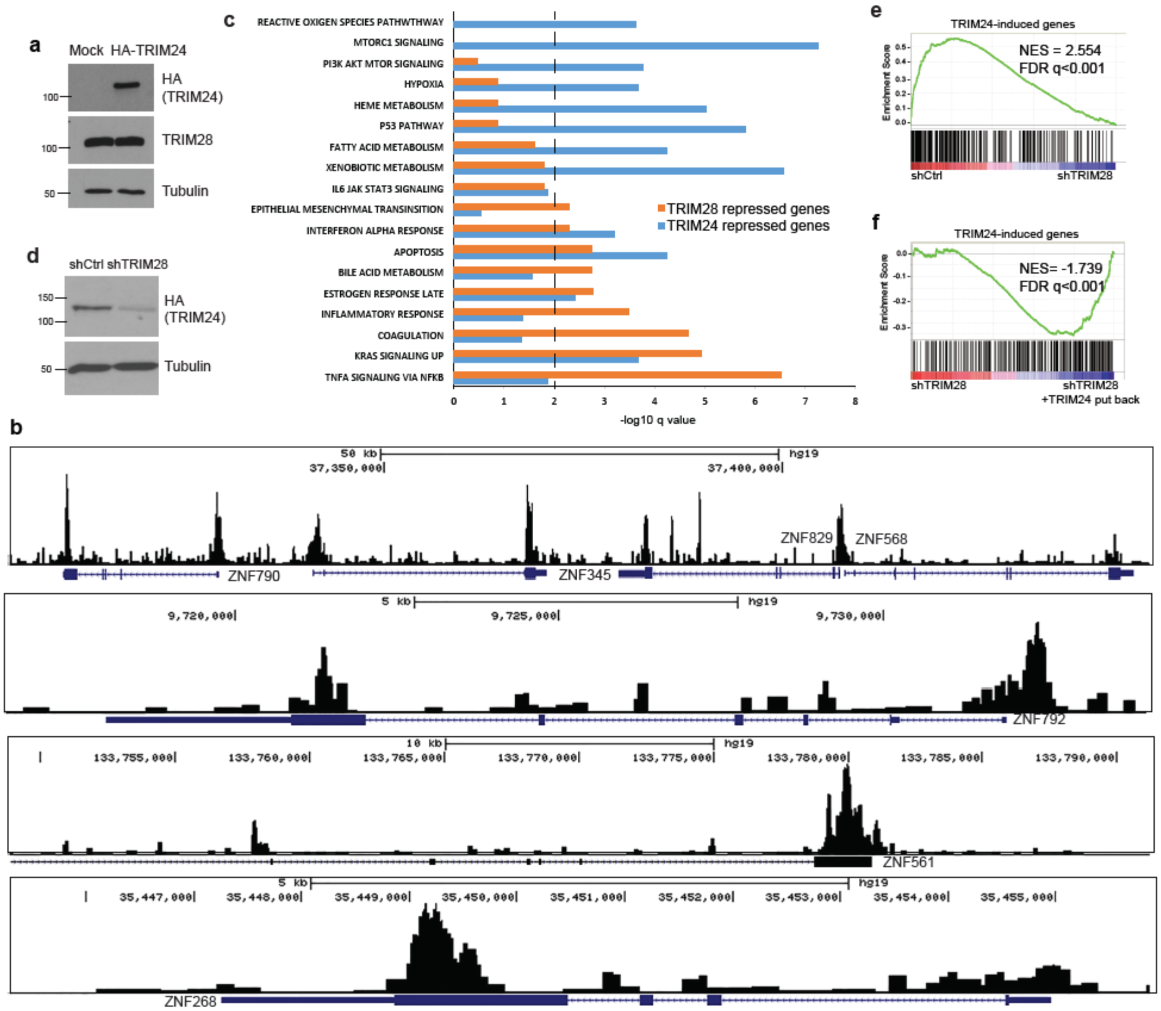

Supplementary Figure 4 (related to Figure 4). TRIM28 co-occupies with TRIM24 on the chromatin to co-regulate many TRIM24 target genes.

(a) Protein lysates were collected from LNCaP cells with stable HA-TRIM24 expression and anti-HA antibody was used to confirm expression of HA-TRIM24 protein.

(b) TRIM28 ChIP-Seq in LNCaP cells. Genome browser tracks depicts TRIM28 occupancy at the promoters and 3' coding exons of zinc finger (ZNF) genes.

(c) HALLMARK gene sets enriched by TRIM28- or TRIM24-repressed genes. TRIM24- or TRIM28-repressed genes were determined by microarray profiling of LNCaP cells expressing pLKO, shTRIM24 or shTRIM28 in the presence of R1881 and subsequently subjected to GSEA analysis of HALLMARK gene sets. The GO-enrichment p values are indicated at the x-axis.

(d) TRIM28 stabilizes HA-TRIM24 protein. LNCaP cells stably expressing HA-TRIM24 were infected with control shRNA or shRNA against TRIM28. Protein lysates were collected and subjected to western blotting analysis.

(e-f) TRIM28 regulates TRIM24-mediated gene expression program. GSEA was performed to determine the enrichment of TRIM24-induced gene sets in expression dataset profiling LNCaP with control or TRIM28 knockdown **(e)**, or with TRIM28 knockdown or TRIM28 knockdown and TRIM24-SFB re-expression **(f)**.

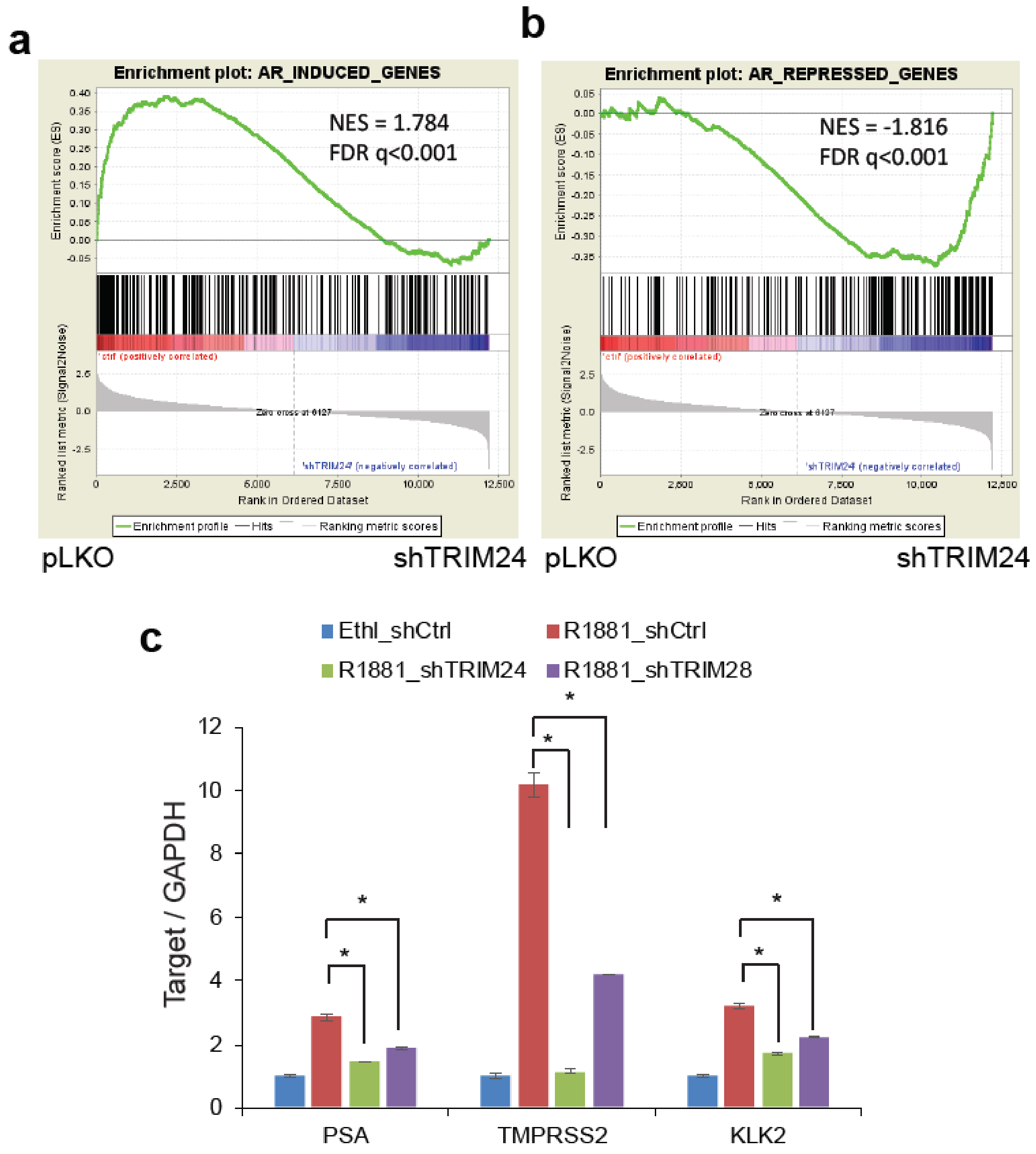

Supplementary Figure 5 (related to Figure 5). TRIM24 positively regulates AR transcriptional program in prostate cancer cells.

(a-b) GSEA was performed to determine the enrichment of AR-induced **(a)** or –repressed gene sets **(b)** in gene expression dataset profiling control and TRIM24-knockdown in androgen-stimulated LNCaP cells.

(c) TRIM28 regulates androgen signaling CRPC. C4-2B cells infected with pLKO, shTRIM24 and shTRIM28 were hormone-starved for 3 days, treated with 1nM R1881 for 24 hours, and subjected to qRT-PCR analysis. Data shown is mean (±SEM, *n*=3). **P* < 0.05 by Student's t-test.

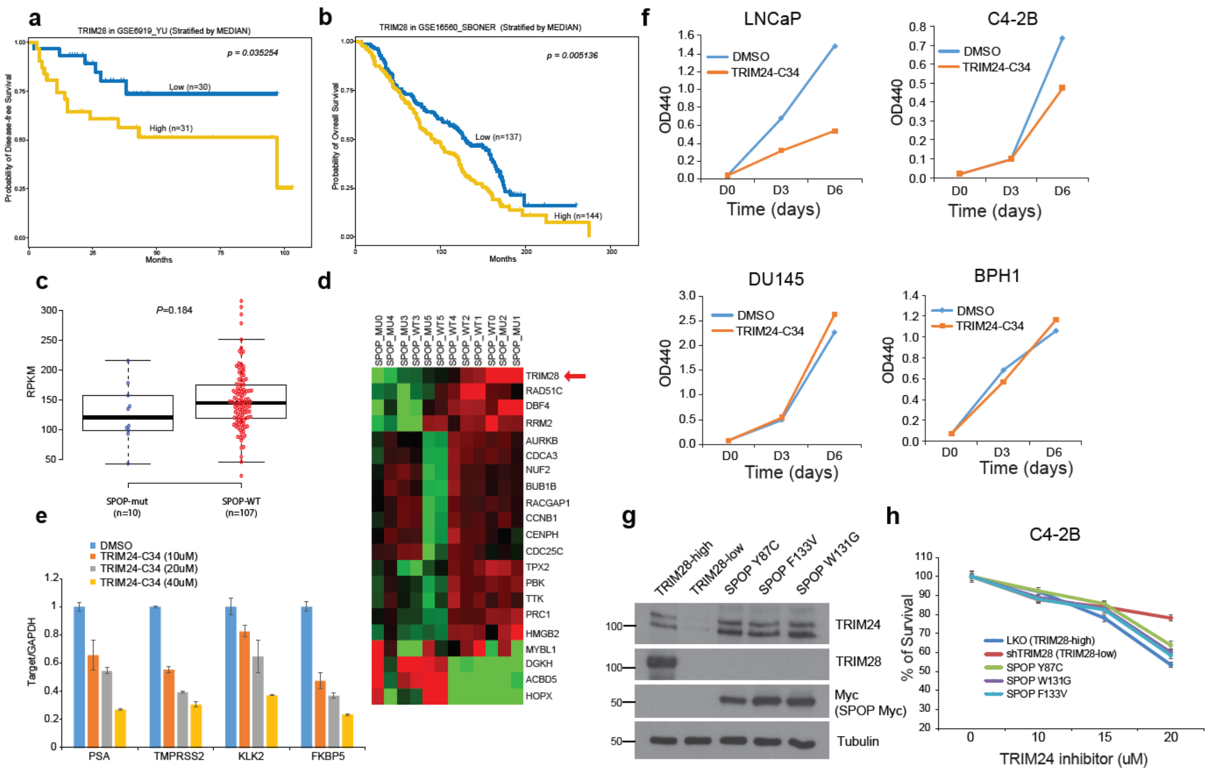

Supplementary Figure 6 (related to Figure 6). High TRIM28 level predicts poor clinical outcome and can be targeted using TRIM24 inhibitor TRIM24-C34.
(a-b) High TRIM28 level is associated with poor clinical outcome of primary PCa. Kaplan-Meier analysis of PCa outcome using the GSE6919 and GSE16560 PCa dataset. Localized PCa cases were stratified based on their TRIM28 expression level and analyzed for biochemical recurrence **(a)** and overall survival **(b)**. The p values for Kaplan-Meier curves were determined using a log-rank test.
(c) TRIM28 expression in SPOP-WT and –Mutant tumors in metastatic CRPC. Boxplot depicts TRIM28 expression in SPOP-WT or –Mutant tumor from SU2C2 RNA-seq dataset. Elements in boxplot: Upper whisker= max value excluding outliers, upper bound of the box=3rd quartile, central line=median, lower bound of the box=1st quartile, lower whisker=least value excluding outliers. Statistical difference was assessed by Student's T-test.
(d) TRIM28 expression correlates to TRIM24-AR coactivated genes in mouse organoid. TRIM28 and TRIM24-AR co-activated gene expression were obtained from RNA-seq dataset of 6 SPOP-WT and –Mutant expressing mouse organoids (Blattner et al., 2017). Heatmap depicts clustered TRIM28 and TRIM24/AR coactivated gene expression.
(e) Compound TRIM24-C34 represses androgen signaling. LNCaP cells were treated with increasing doses (0, 10, 20, 40µM) of TRIM24-C34 for 4 days and mRNA was then extracted and subjected to qPCR analysis using corresponding primer sets and normalized to GAPDH. Data shown is mean (±SEM, *n*=3).
(f) TRIM24-C34 inhibits cell growth in AR-positive but not AR-negative prostate cancer cell lines. BPH1, DU145, LNCaP and C4-2B were cultured in the presence of 10µM compound. WST1 assay was performed at day 0, 3 and 6 to evaluate the cell proliferation.

(g-h) CRPC with SPOP mutation or TRIM28 elevation exhibits sensitivity to TRIM24 inhibitors. Expression of Myc-SPOP-Y87C/F133V/W131G and various proteins were confirmed by western blotting **(g)**. These cells were then treated with increasing doses of TRIM24-bromodomain inhibitor (TRIM24-C34) for 14 days and measured for cell survival in colony formation assays **(h)**.

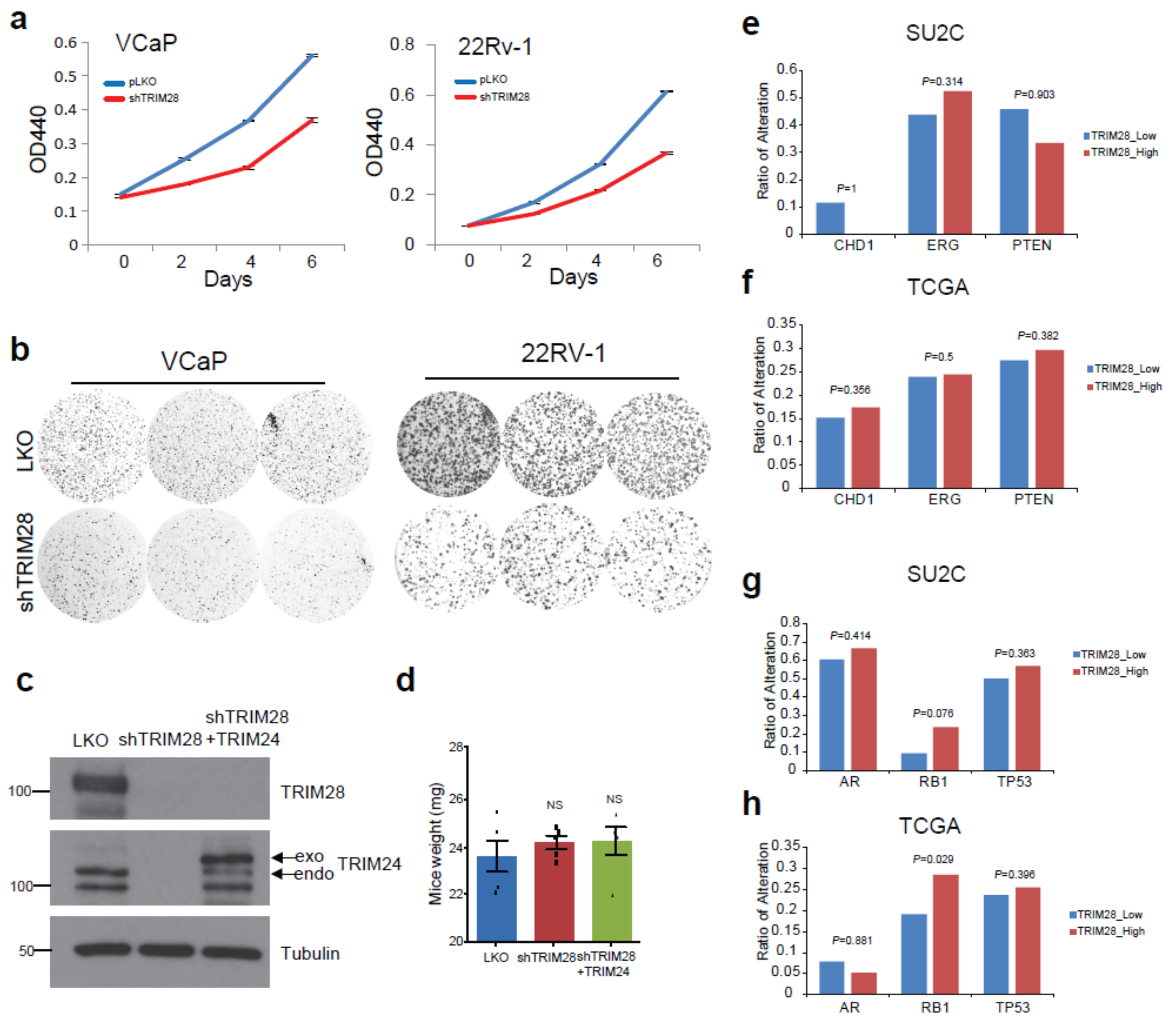

Supplementary Figure 7 (related to Figure 7). TRIM28 promotes prostate cancer growth.
(a-b) Proliferation of control or TRIM28-knockdown VCaP and 22Rv-1 cells were evaluated by WST-1 assay **(a)** and colony formation assay **(b)**.
(c) Protein lysates from C4-2B was subjected to western blotting analysis.
(d) Mice weight was plotted as a bar graph. Data shown is mean (±SEM, *n*=5). NS: not significant.
(e-f) TRIM28 overexpressing is not accompanied with CHD1, ERG and PTEN alteration. Tumors of TRIM28-High (ranked top 20% in TRIM28 expression) or -Low (remaining 80%) from SU2C RNA-seq dataset **(e)** or TCGA dataset **(f)** were examined for CHD1, ERG and PTEN alteration. The probability of genomic alteration was plotted as bar graph, and Statistical difference was assessed by Student's T-test.
(g-h) TRIM28 overexpressing is associated with RB1 loss. The probabilities of genomic alteration of AR, RB1 and TP53 in TRIM28-High or -Low tumors from SU2C RNA-seq dataset **(g)** or TCGA dataset **(h)** were plotted as bar graph, and Statistical difference was assessed by Student's T-test.

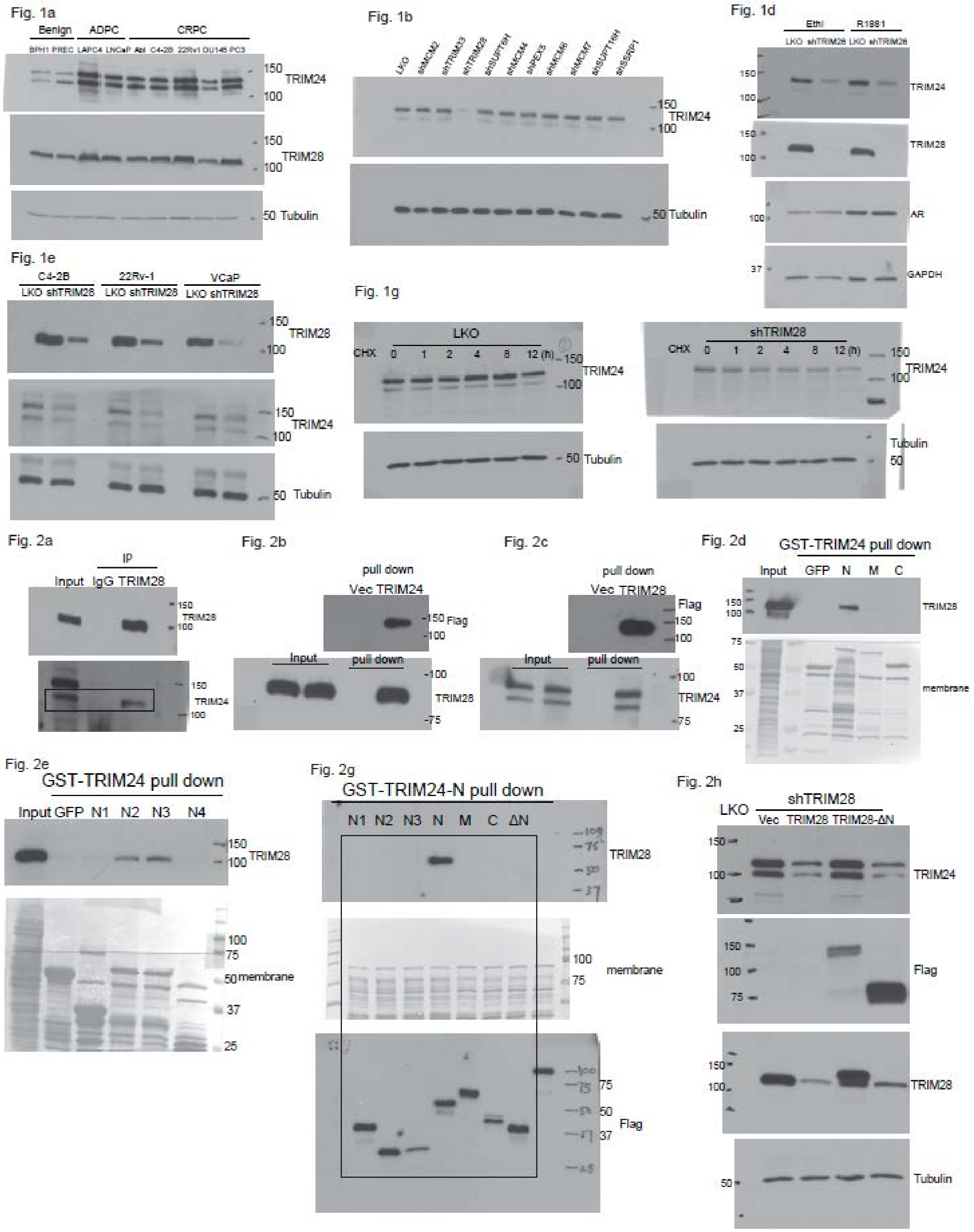

Supplementary Figure 8. Unprocessed blot images for the western results in Figure 1-2.

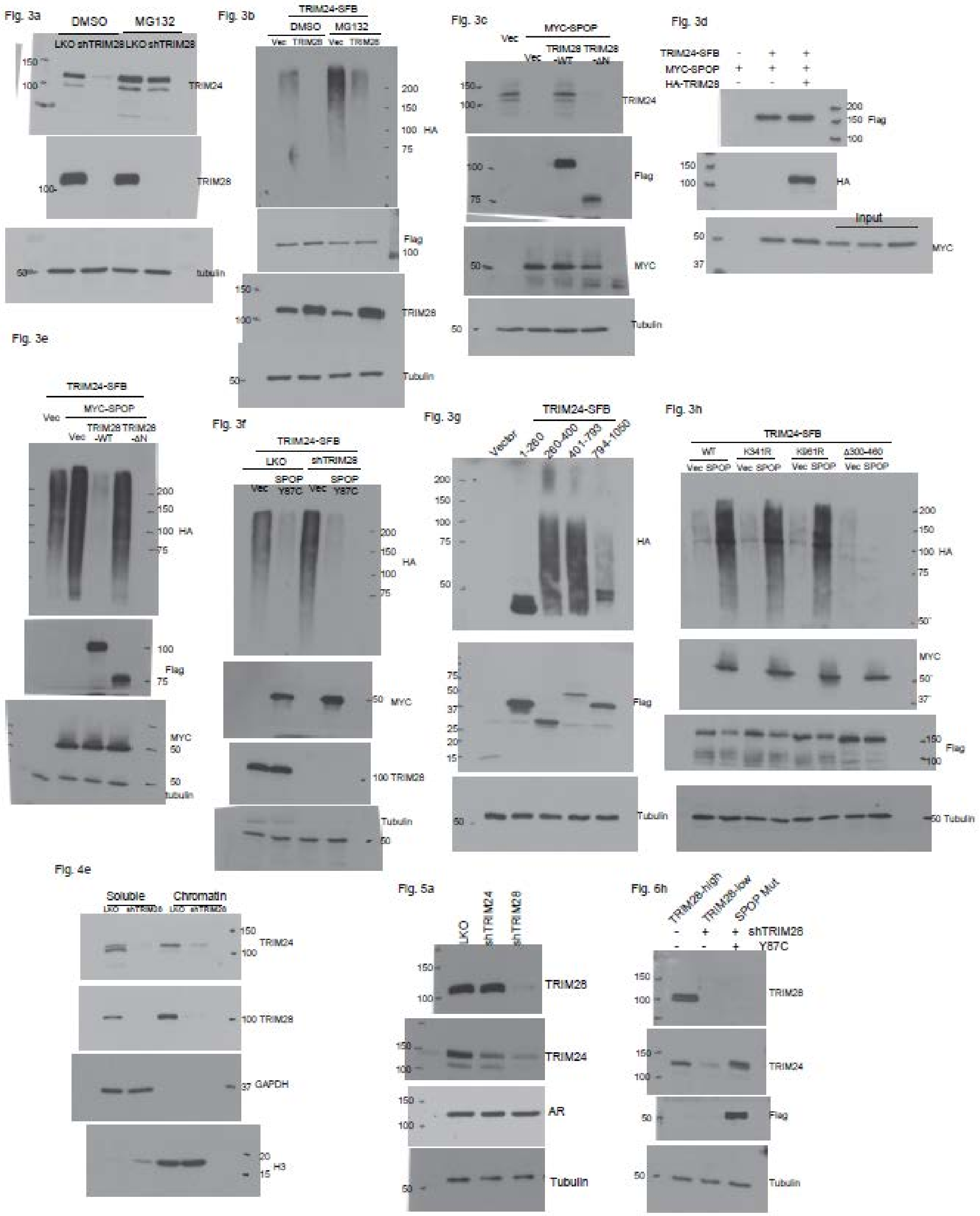

Supplementary Figure 9. Unprocessed blot images for the western results in Figure 3-6

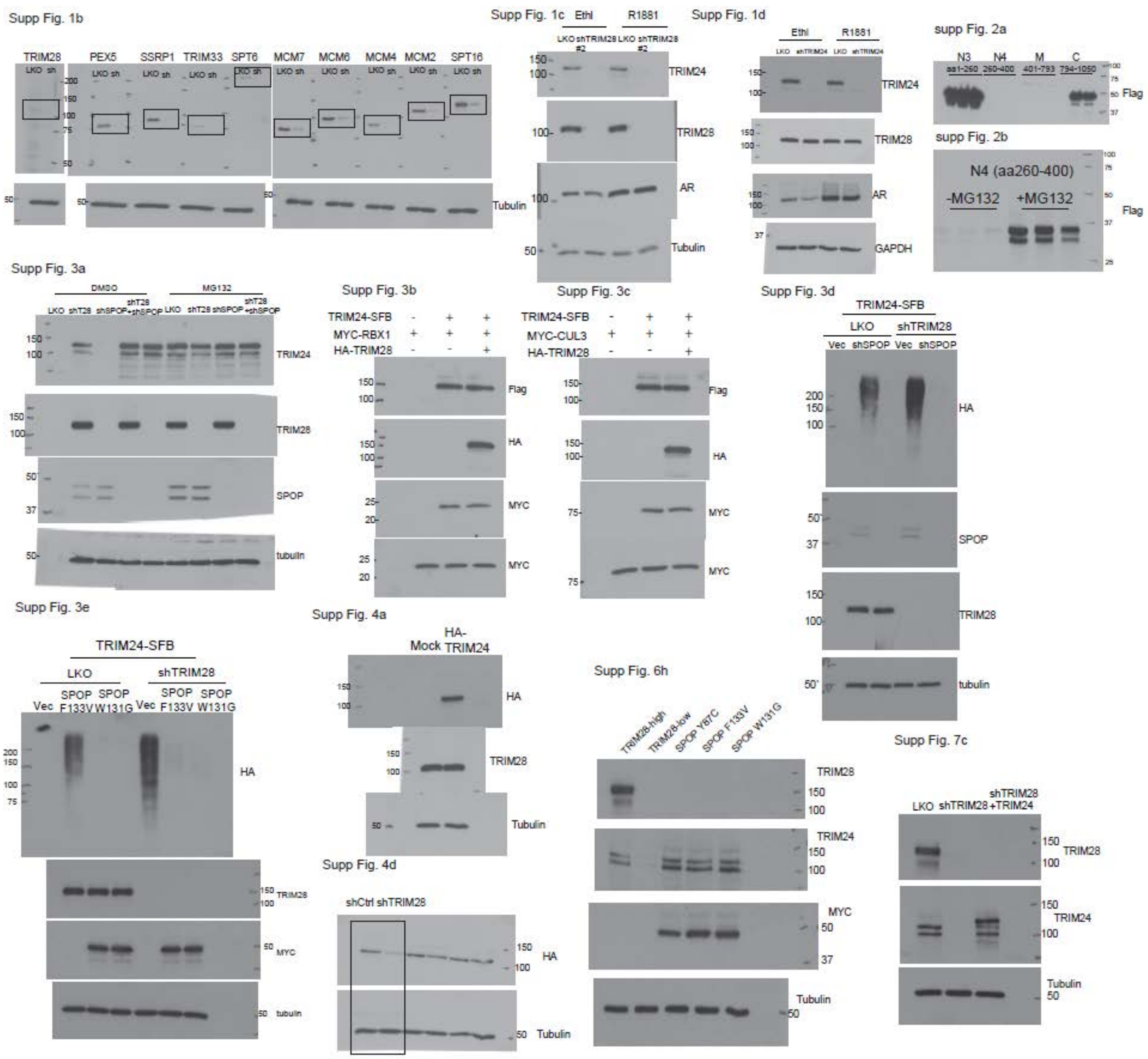

Supplementary Figure 10. Unprocessed blot images for the western results in supplementary figures

Supplementary Table 1: Top TRIM24 interactors

Protein Name	Unique peptide	Total peptide	Mw
TRIM24	76	843	116.76
MCM2	29	42	101.83
TRIM33	28	41	122.46
TRIM28	20	29	88.49
SUPT6H	20	23	198.95
MCM4	19	28	96.5
PEX5	18	20	70.82
MCM6	15	16	92.83
MCM7	13	14	81.26
SUPT16H	11	11	119.84
SSRP1	11	11	81.02
SPOP	4	4	42.1

Supplementary Table 2: Antibody used in this study

Antibody	Source	Catalog Number	Dilution	Lot no
Rabbit Anti-TRIM28	Abcam	ab10483	1:2000	GR17300-19
Rabbit Anti-Histone H3	Abcam	ab1791	1:200,000	183115
Rabbit Anti-HA	Abcam	ab9110	1:5000	GR261166-6
Rabbit Anti-TRIM24	Bethyl Laboratory	A300-815A	1:1000	A300-815A-2
Goat Anti-Myc (A-14)	Santa Cruz	sc-789-G	1:2000	NA
Rabbit Anti-Ki67 (D2H10)	Cell signaling	9027	1:200	6
Rabbit Anti-Flag	Sigma	F7425	1:2000	086M4803V
Mouse Anti-Flag M2	Sigma	F1804	1:2000	SLBV9325
Mouse Anti-MCM2 (E-8)	Santa Cruz	sc-373702	1:500	H1117
Mouse Anti-TIF1γ (XX-19)	Santa Cruz	sc-101179	1:500	D0318
Mouse Anti-SPT16 (D-3)	Santa Cruz	sc-377028	1:500	B1717
Mouse Anti-SPT6 (E-4)	Santa Cruz	sc-393920	1:500	H2417
Mouse Anti-SSRP1 (D-7)	Santa Cruz	sc-74536	1:500	F0717
Mouse Anti-MCM4 (G-7)	Santa Cruz	sc-28317	1:500	I0717
Mouse Anti-Peroxin 5 (B-3)	Santa Cruz	sc-137103	1:500	G2717
Mouse Anti-MCM6 (H-8)	Santa Cruz	sc-393618	1:500	L1517
Mouse Anti-MCM7 (141.2)	Santa Cruz	sc-9966	1:500	A0818
Mouse Anti-GAPDH (G-9)	Santa Cruz	sc-365062	1:5000	K2316
Mouse Anti-α-Tubulin (DM1A)	Santa Cruz	sc-32293	1:5000	C0818
Rabbit AR antibody	Santa Cruz	sc-816x	1:2000	J216
Rabbit SPOP antibody (N1C1)	Genetex	GTX106942	1:1000	40073

Supplementary Table 3: Oligonucleotides that were utilized in this study

**Name**	**Sequence**	**Application**
TRIM24 F	TGTGAAGGACACTACTGAGGTT	qRT-PCR
TRIM24 R	GCTCTGATACACGTCTTGCAG	qRT-PCR
GAPDH F1	TGCACCACCAACTGCTTAGC	qRT-PCR
GAPDH R1	GGCATGGACTGTGGTCATGAG	qRT-PCR
PSA F1	ACGCTGGACAGGGGGCAAAAG	qRT-PCR
PSA R1	GGGCAGGGCACATGGTTCACT	qRT-PCR
KLK2 F1	CCATGCCTGGAGACATATCA	qRT-PCR
KLK2 R1	TCCAGCACATGTCACTCTCC	qRT-PCR
TMPRSS2 F1	CAGGAGTGTACGGGAATGTGATGGT	qRT-PCR
TMRPSS2 R1	GATTAGCCGTCTGCCCTCATTTGT	qRT-PCR
AURKB F1	TTTGCTATGAGCTGCTGGTG	qRT-PCR
AURKB R1	CACAGAAGCGGGGAACTTTA	qRT-PCR
PBK F1	CCAAACATTGTTGGTTATCGTGC	qRT-PCR
PBK R1	GGCTGGCTTTATATCGTTCTTCT	qRT-PCR
TPX2 F1	ATGGAACTGGAGGGCTTTTTC	qRT-PCR
TPX2 F2	TGTTGTCAACTGGTTTCAAAGGT	qRT-PCR
KIAA0066 pF1	CTAGGAGGGTGGAGGTAGGG	ChIP-PCR
KIAA0066 pR1	GCCCCAAACAGGAGTAATGA	ChIP-PCR
PSA pF1	GCCTGGATCTGAGAGAGATATCATC	ChIP-PCR
PSA pR1	ACACCTTTTTTTTTCTGGATTGTTG	ChIP-PCR
KLK2 pF1	AGCATCTAGGTGCCAACAGG	ChIP-PCR
KLK2 pR1	GACAAGGCGATGGAGAGAAC	ChIP-PCR
TMPRSS2 pF1	GGTAAACTCTCCCTGCCACA	ChIP-PCR
TMPRSS2 pR1	TACTCCAGGAAGTGGGGATG	ChIP-PCR
AURKB pF1	CGGCGGTTTTGTTATTGG	ChIP-PCR
AURKB pR1	CTCGGCCTCTGTGTTCGAT	ChIP-PCR
PBK pF1	AGCTGCCTCTAGCACCAACAC	ChIP-PCR
PBK pR1	CAGGAGGGTTCGAATTGCAA	ChIP-PCR
TPX2 pF1	GGCTTCTCGAGCTCACTAGG	ChIP-PCR
TPX2 pR1	CCCTCCTCTCAGGAAGGTCT	ChIP-PCR
TRIM24 1 FP	ATGGAGGTGGCGGTGGAGAAG	Cloning
TRIM24 1050 RP	TTTAAGCAACTGGCGTTCTTC	Cloning
TRIM28 1 FP	ATGGCGGCCTCCGCGGCGGCAGCC	Cloning
TRIM28 835R	GGGGCCATCACCAGGGCCACCAG	Cloning
TRIM28 250 RP	CCTCACTGCATCCTCTAAGAACTG	Cloning
TRIM28 400 RP	CTTGGTCCAGGCATTGAGGTCCCA	Cloning
TRIM28 380 FP	ATGGTGGATCCCGTGGAGCCACATGGC	Cloning
TRIM28 628 RP	GCAAATGGTGGCACTGTCATCCAG	Cloning
TRIM28 602 FP	ATGGGGCTGGAGGTGGTGGCTCCTGAG	Cloning
TRIM28 250 FP	ATGTTAGAGGATGCAGTGAGGAACCAG	Cloning
TRIM28 150 RP	GTTCGCATCCTGGGCGTCGGT	Cloning

TRIM24 RP HA	TCAAGCGTAATCTGGAACATCGTATGGGTAAGC GTAATCTGGAACATCGTATGGGTA TTTAAGCAACTGGCGTTCTTC	Cloning
TRIM24 F1F BamH1	GCG GGATCC ATGGAGGTGGCGGTGGAGAAG	Cloning
TRIM24 F1R NOT1	AGCT GCGGCCGC GGTCACTGGGGATGCATCACA	Cloning
TRIM24 F2F BamH1	GCG GGATCC ATG AACAACACCATCCAATTTCACTG	Cloning
TRIM24 F2R NOT1	AGCT GCGGCCGC GTGAAGTCCAGGTTGATCTCC	Cloning
TRIM24 F3F Sal1	GCG GTCGACAG ATG CAGGACAATTCCTCAAATGGA	Cloning
TRIM24 F3R NOT1	AGCT GCGGCCGC TTTAAGCAACTGGCGTTCTTC	Cloning
TRIM24 100R NOT1	AGCT GCGGCCGC CTCGGCCGAGCCCAGCATGGG	Cloning
TRIM24 100F BamH1	GCG GGATCC ATG GAGACCCCGCCACCCGTCCCT	Cloning
TRIM24 260R NOT1	AGCT GCGGCCGC TTCTATAAATTGGTATCTATG	Cloning
TRIM24 260F BamH1	GCG GGATCC ATG ATG GAAGAAGCTTTTCAGAATCAG	Cloning
SPOP CF	ATGTCAAGGGTTCCAAGTCCT	Cloning
SPOP CR	GGATTGCTTCAGGCGTTTGCG	Cloning
SPOP Y87C FP	AGCAAAGATTACCTGTCACTTTGCCTGTTACTG GTCAGCTGTCCA	Cloning
SPOP Y87C RP	TGGACAGCTGACCAGTAACAGGCAAAGTGACA GGTAATCTTTGCT	Cloning
SPOP F133V FP	GGTTTGTGCAAGGCAAAGACGGGGGATTCAAG AAATTCATCCGT	Cloning
SPOP F133V RP	ACGGATGAATTTCTTGAATCCCCCGTCTTTGCCT TGCACAAACC	Cloning
SPOP W131G FP	GCAAGGCAAAGACTGGGGAGTCAAGAAATTCA TCCGTAGAGATT	Cloning
SPOP W131G RP	AATCTCTACGGATGAATTTCTTGACTCCCCAGTC TTTGCCTTGC	Cloning
TRIM24 K341R FP	CTTGCAAAGGACCATCGCATGAGACTTATGCAA CAACAACAGGAA	Cloning
TRIM24 K341R RP	TTCCTGTTGTTGTTGCATAAGTCTCATGCGATGG TCCTTTGCAAG	Cloning
TRIM24 K961R FP	GAAGATTATTCCATGTACTCAAGACCTGAAGAT TTTGTAGCTGAT	Cloning
TRIM24 K961R RP	ATCAGCTACAAAATCTTCAGGTCTTGAGTACAT GGAATAATCTTC	Cloning
TRIM24 del 300-460aa FP	AACAGAATTATTGAAGTAAATCAAACACAGATC AGCCTAGCTCAATTA	Cloning

TRIM24 del 300-460aa RP	TAATTGAGCTAGGCTGATCTGTGTTTGATTTACTTCAATAATTCTGTT	Cloning

Supplemental Table 4: shRNA used in this study

Target	Supplier	Catalog number
TRIM28-1	sigma	TRCN0000199141
TRIM28-2	sigma	TRCN0000017998
TRIM24	sigma	TRCN0000021260
MCM2	sigma	TRCN0000278310
TRIM33	sigma	TRCN0000322655
SUPT6H	sigma	TRCN0000019732
MCM4	sigma	TRCN0000074244
shSUPT16H	sigma	TRCN0000293350
shSSRP1	sigma	TRCN0000343894
shPEX5	sigma	TRCN0000330702
shMCM6	sigma	TRCN0000275649
shMCM7	sigma	TRCN0000330416
shSPOP	Sigma	TRCN0000139181
