# Supplementary material for: TRIM28 protects TRIM24 from SPOP-mediated degradation and promotes prostate cancer progression
Source: Nat Commun. 2018 Nov 27;9:5007. doi: 10.1038/s41467-018-07475-5 (PMC6258673; doi:10.1038/s41467-018-07475-5)
Supplement: Supplementary file 4 — Description of Additional Supplementary Files [file 41467_2018_7475_MOESM4_ESM.doc]

**Title:** Supplementary Data 1
**Description:** Sequence motifs enriched in TRIM24 or TRIM28 ChIP-seq binding sites. All high-throughput data, including microarray and ChIP-seq, have been deposited to GEO (GSE108146).
